# Mental health problems among hotline callers during the early stage of COVID-19 pandemic

**DOI:** 10.7717/peerj.13419

**Published:** 2022-05-23

**Authors:** Jing An, Yi Yin, Liting Zhao, Yongsheng Tong, Nancy H. Liu

**Affiliations:** 1 Beijing Huilongguan Hospital, Beijing, Beijing, China; 2 Peking University HuiLongGuan Clinical Medical School, Beijing, China; 3 WHO Collaborating Center for Research and Training in Suicide Prevention, Beijing, China; 4 Department of Psychology, University of California, Berkeley, Berkeley, CA, United States of America

**Keywords:** COVID-19 Pandemic, Hotline, Mental health

## Abstract

**Background:**

The study aims to explore the mental health of the hotline callers during the COVID-19 pandemic in China.

**Methods:**

Callers (*N* = 10,490) from the Beijing Psychological Support Hotline from January 21st to June 30th in 2019 and 2020 were enrolled and divided into two groups (during (2020) and before (2019) COVID-19 pandemic). The severity of depressive symptoms, psychological distress, hopefulness, and suicidal ideation (SI) was assessed. Demographic characteristics and major concerns were also collected. Mann-Whitney U and chi-square test were used to compare the differences in mental health conditions and major concerns between two years and between different age groups. The multivariable logistic regression was used to explore whether mental health conditions were associated with pandemic and demographic factors.

**Results:**

Results from multivariable logistic regression analysis indicated that the change in suicidal ideation (OR = 1.52, 95% CI: 1.21–1.92) was significantly different across age groups. Callers during the pandemic reported a higher level of hopefulness (OR = 1.13, 95% CI [1.03–1.24]), a lower level of depressive symptoms (OR = 0.81, 95% CI [0.74–0.89]) and psychological distress (OR = 0.89, 95% CI [0.81–0.98]), and were less likely to report SI (OR = 0.69, 95% CI [0.61–0.77]) compared with callers before the pandemic.

**Conclusions:**

Compared with callers before the pandemic, hotline callers during the early stage of COVID-19 pandemic did not present significant mental health problems. Younger callers during the pandemic were more vulnerable for the presence of suicidal ideation. Hotline-based crisis interventions might provide specific psychological support to cope with troubles during the pandemic.

## Introduction

The coronavirus disease 2019 (COVID-19) pandemic poses a significant threat to the mental health in the general population (Cooke et al., 2020; Debowska et al., 2020; Brooks et al., 2020; Shi et al., 2020; Luo et al., 2020). Anxiety, depression, stress, and sleep problems are commonly reported among the public during the pandemic (Cooke et al., 2020; Debowska et al., 2020; Brooks et al., 2020; Shi et al., 2020; Luo et al., 2020). During the pandemic, higher suicidal risk at population level was one of the most important concerns, given people were widely exposed to social isolation, financial recession, and barriers to accessing mental health care (Gunnell et al., 2020). Previous online surveys found that 4.6% to 17.5% of individuals reported suicide ideation (SI), 1.2% to 4.9% had attempted suicide in the last month (Ammerman et al., 2021; Bryan, Bryan & Baker, 2020), and 7.6% of the respondents were found to have higher suicide risk during the pandemic (Caballero-Dominguez, Jimenez-Villamizar & Campo-Arias, 2020). Previous epidemics, as Severe Acute Respiratory Syndromes (SARS) in 2003 has also shown to be associated with the increase in suicide (Zortea et al., 2020).

Individuals in different age groups seem to be impacted psychologically in different tendencies during the pandemic. Previous evidence suggests a relatively higher severity of mental health problems, such as anxiety and depression in younger adults during the COVID-19 pandemic (Huang & Zhao, 2020; Liu et al., 2020a; Liu et al., 2020b; Jia et al., 2020). Younger adults have the highest level, while the older have the lowest level of SI during the pandemic (O’Connor et al., 2020; Czeisler et al., 2020; Iob, Steptoe & Fancourt, 2020). However, the evidence from the SARS epidemic suggested that the suicide rate increased greatly among older adults in Hong Kong (Cheung, Chau & Yip, 2008; Chan et al., 2006).

The rapid transmission of COVID-19 led to a halt in delivering face-to-face psychological services, and as a result, online mental health service resources, such as the internet and telephone hotlines were widely used to provide psychological assessment and interventions (Feinstein et al., 2020; Wang, Wei & Zhou, 2020; Liu et al., 2020a; Liu et al., 2020b). Hotlines, in particular, have become a popular way for individuals seeking for psychological help during COVID-19. Beijing Psychological Support Hotline (BPSH) is a national 24-hour toll-free crisis hotline in China, and provides psychological services for hundreds of thousands of callers every year. More than half of the BPSH callers reported SI before the pandemic (Zhao et al., 2021). Considering the fact that more individuals exposed to the suicide risk factors during the pandemic, it is vital to understand whether mental health problems of hotline callers among different age groups increased during the pandemic, in order to guide decisions on further effective allocation of psychological service resources in the hotline.

The present study compared the mental health status of the BPSH callers between the callers before the COVID-19 pandemic (the first half-year of 2019) and during the pandemic (the first half of year of 2020). The findings of our study would be helpful to discuss whether the impacts of the COVID-19 pandemic on the mental health of hotline callers. It will contribute to the development of hotline-based psychological interventions during public health emergencies in the future.

## Materials & Methods

### Subjects

The study was conducted at the BPSH, which provided psychological intervention for Mandarin-speaking callers before and during the COVID-19 pandemic. COVID-19 was confirmed as a human-to-human transmitted virus on January 20th, 2020, and remained a national epidemic in mainland China until summer 2020. Thus, in the present study, callers from January 21st to June 30th, 2020 were enrolled as participants during the early stage of the pandemic. In addition, callers in the same time period in 2019 were enrolled as pre-pandemic participants.

A total of 25,239 calls were answered in that periods of 2019 and 2020 in the BPSH. Exclusion criteria were: (1) the caller’s main purpose was not seeking psychological services (*e.g.*, only asking for information), (2) “null” calls (i.e., silence, harassing, or hoax calls), or (3) calls lasting for less than 10 min. For repeat calls from the same caller, only the first call was included in the analysis. Finally, 10,490 calls were enrolled into the study (4,940 in 2019, accounting for 47.1% and 5,550 in 2020, accounting for 52.9%).

The study was approved by the Institutional Review Board of Beijing Huilongguan Hospital (2020-19-Science). Each caller was informed by a voice message that the call would be tape-recorded, data would be collected, deidentified, and analyzed.

### Measurements

In the BPSH, mental health factors included severity of depression, psychological distress, level of hopefulness, and the presence of SI (Bell et al., 2015; Walker, McGee & Druss, 2015; Davidson et al., 2009). For depression, a structured assessment questionnaire (Phillips et al., 2007) was used to determine the severity and duration of each of nine depressive symptoms in the last 2 weeks prior to the index call. The assessment has excellent inter-rater reliability (ICC = 1.00), test-retest reliability (ICC = 0.91) and consistency with Structured Clinical Interview for DSM-IV (SCID), (Kappa = 0.87) (Li et al., 2007). The score was calculated by the item severity multiplied by duration of the symptom. The total score of depressive symptoms was converted into 0 to 100, with a higher score indicating more severe depressive symptoms.

Psychological distress was assessed by asking callers “To what extent do you feel psychological distressed?”. Callers were instructed that a score of 0 means completely no psychological distress and 100 means feeling completely distressed (Tong et al., 2020). Hopefulness was assessed by asking callers “To what extent do you feel hopeful?”. Callers were instructed that a score of 0 means absolutely hopeless and 100 means completely hopeful (Tong et al., 2020).

SI was assessed by asking the caller “Have you repeatedly thought about taking your life or hurting yourself in the last two weeks?” or “Have you felt too tired and without meaning to continue to live in the last two weeks?”. If a caller responded “yes” to any of the above two questions, he/she would be regarded as having suicidal ideation (Tong et al., 2020).

During the routine counselling of hotline, the operator and the caller would work together on the identification and coping with major concern of the caller. The identification of the major concern was based on whether the caller had a history of being diagnosed as psychiatric illness or receiving outpatient or inpatient psychiatric treatments (medication, psychotherapy, and other therapies such as ECT), caller’s complaints on psychological disturbance or life events, and severity of depression assessed by structured questionnaire. The concerns were categorized into seven categories or sources of distress: (1) family relationship problems, *e.g.*, conflicts with family members; (2) non-family relationship problems, *e.g.*, interpersonal conflicts with non-family members, such as friends and colleagues; (3) financial problems, *e.g.*, debts, failed investments, *etc.*; (4) work-related problems, *e.g.*, work pressure, losing the job, setback in career advancement; (5) clinically significant depression, based on a structured assessment (Phillips et al., 2007); (6) other negative life events, *e.g.*, caller was distressed by events other than the five categories listed above; (7) other psychiatric problems, *e.g.*, caller reported a history of any mental disorder other than depression.

The demographic characteristics were collected by the hotline counsellor at the beginning of answering the call.

### Statistical analysis

The final sample of callers was split into two groups, namely, calls during the pandemic (January–June 2020) and before the pandemic (January–June 2019). Mann–Whitney U and chi-square tests were used to compare the differences in mental health conditions and major concerns of callers between two years and different age groups. The multivariable logistic regression was used to explore whether mental health conditions were associated with demographic factors and the pandemic. Gender, marital status, education levels, age group, year of index call (2019 and 2020), and an interaction term between age group by before/during pandemic (namely, year of index call) were entered into the model to test whether the effect of age groups on mental health conditions was different between the two pandemic periods (before/during) (Altman & Bland, 2003). Dependent variables included depression and psychological distress severity, level of hopefulness, and SI. All dependent variables were dichotomized and analyzed separately. The score for the hopefulness, psychological distress, and depressive symptoms were respectively dichotomized by the median scores, namely, 30, 80, 68. Data analyses were conducted using SPSS 18.0.

## Results

**Figure 1:**
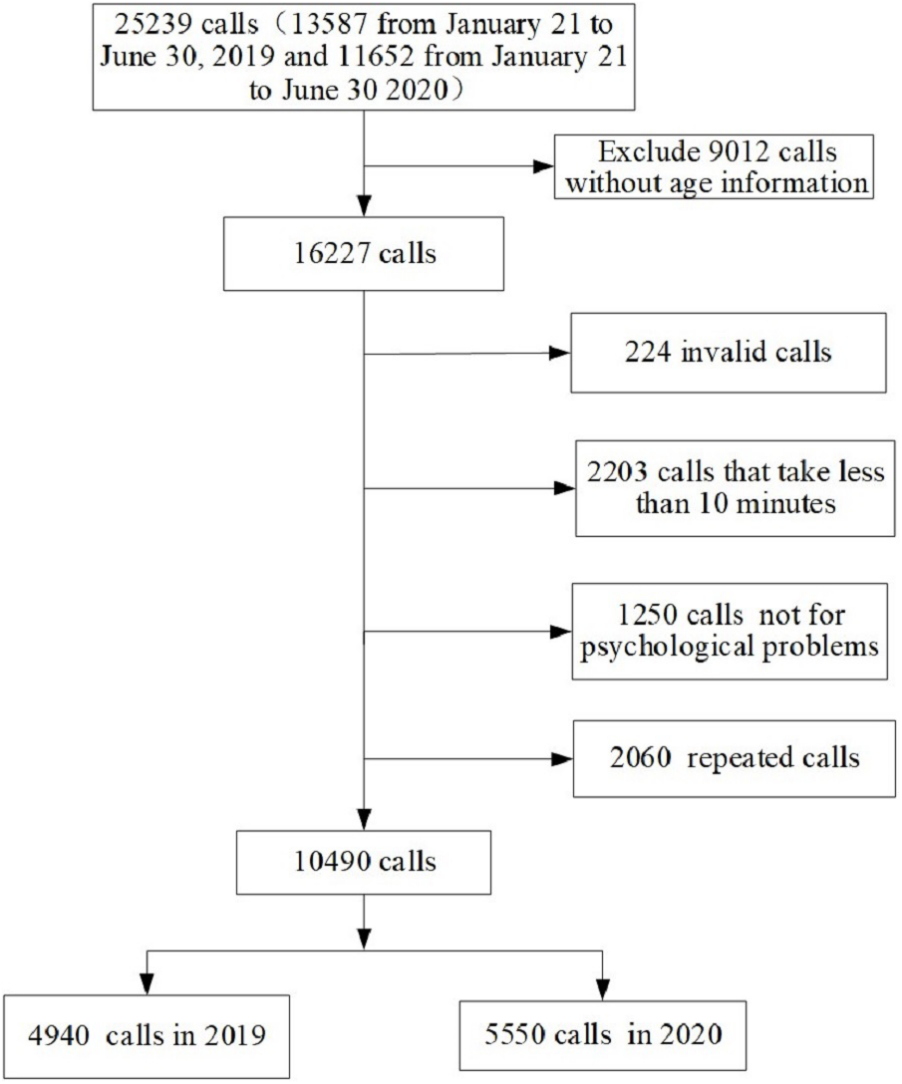
Flow chart for recruitment of final sample.

A total of 10,490 calls from 2019 January 21st to June 30th and in the same period in 2020 were included in the final data analysis. A detailed overview of enrolling and screening hotline callers is shown in Fig. 1. As seen in Table 1, there was no significant difference in marital status between callers before and during pandemic (*p* = 0.070), however, callers during the COVID-19 pandemic were more likely to be female (*p* < 0.001), over 30 years (*p* = 0.048) and highly educated (*p* < 0.001) than callers before the pandemic.

**Table 1:** Characteristics of callers before and during COVID-19 pandemic.

Characteristics	Before pandemic (*n* = 4940) *n* (%)	During pandemic (*n* = 5550) *n* (%)	*χ* ^2^	*p*
Gender			66.14	0.000
Female	2604 (52.7)	3364 (60.6)		
Male	2334 (47.3)	2186 (39.4)		
Age group			3.91	0.048
<30 years old	3756 (76.0)	4127 (74.4)		
≥30 years old	1184 (24)	1423 (25.6)		
Marital status			3.29	0.070
Unmarried	3791 (76.7)	4175 (75.2)		
Married	1149 (23.3)	1375 (24.8)		
Education years			41.99	0.000
0–9	657 (13.3)	700 (12.6)		
10–12	1417 (28.7)	1307 (23.5)		
≥13	2866 (58.0)	3543 (63.8)		

**Notes.**

Each variable contains missing values, so the sum of the callers of each variable is less than the total number of callers.

Callers before the pandemic were more likely to report SI (78.7% *vs*. 73.4%, *χ*^2^=31.29, *p* < 0.001), higher depression score (*Z* =  − 3.57, *p* < 0.001), lower level of hopefulness (*Z* =  − 3.03, *p* = 0.002) than callers during pandemic (Table 2). Callers during the pandemic were more likely to endorse depression problems (25.6% *vs* 21.3%, *χ*^2^ = 26.49, *p* < 0.001) and family relationship problems (23.2% *vs* 20.1%, *χ*^2^ = 14.85, *p* < 0.001), and less likely to encounter other negative events (5.7% *vs* 7.0%, *χ*^2^ = 6.94, *p* = 0.008), financial problems (8.0% *vs* 10.3%, *χ*^2^ = 16.24, *p* < 0.001), and relationship problems (28.1% *vs* 30.3%, *χ*^2^ = 5.69, *p* = 0.017) than callers before the pandemic (Table 2).

**Table 2:** Comparison of mental health status and concerns of hotline callers between different age groups and years.

**Variables**	Before pandemic (*n* = 4940)	During pandemic (*n* = 5550)		Younger (*n* = 7883)	Older (*n* = 2607)	
	**Median (IQR)**	**Median (IQR)**	***Z***	**Median (IQR)**	**Median** **(IQR)**	***Z***
Hopefulness	30 (2,50)	30 (5,50)	−3.03^**^	30 (5,50)	35 (5,60)	−5.03^***^
Psychological distress	80 (70,95)	80 (70,95)	−1.82	80 (70,95)	80 (70,95)	−1.33
Depression severity	69 (53,81)	67 (52,80)	−3.57^***^	68 (53,81)	67 (49,81)	−2.92^**^
	**n (%)**	**n (%)**	*χ* ^**2**^	**n (%)**	**n (%)**	*χ* ^**2**^
Presence of suicidal ideation	2909 (78.7)	3165 (73.4)	31.29^***^	4816 (78.9)	1258 (66.1)	130.66^***^
	**n (%)**	**n (%)**	*χ* ^**2**^	**n (%)**	**n (%)**	*χ* ^**2**^
Family relationship problems	992 (20.1)	1287 (23.2)	14.85^***^	1525(19.3)	754 (28.9)	105.66^***^
Non-family relationship problems	1495 (30.3)	1562 (28.1)	5.69^*^	2646 (33.6)	411 (15.8)	300.62^***^
Financial problems	509 (10.3)	446 (8.0)	16.24^***^	606 (6.7)	349 (13.4)	76.91^***^
Work problems	344 (7.0)	367 (6.6)	0.51	530 (6.7)	181 (6.9)	0.15
Depression	1052 (21.3)	1419 (25.6)	26.49^***^	1846(23.4)	625 (24)	0.34
Other negative events	345 (7.0)	318 (5.7)	6.94^**^	414 (5.3)	52 (9.6)	61.16^***^
Psychiatric problems	833 (16.9)	906 (16.3)	0.55	1278 (16.2)	461 (17.7)	3.07

**Notes.**

* *p* < 0.05.

** *p* < 0.01.

*** p <0.001.

Each variable contains missing values, so the sum of the callers of each variable is less than the total number of callers.

There were also significant differences between the two age groups in mental health status, specifically in hopefulness (*Z* =  − 5.03, *p* < 0.001), the severity of depressive symptoms (*Z* =  − 2.92, *p* = 0.004), presence of SI (*χ*^2^ = 130.66, *p* < 0.001) (Table 2). Proportions of major concerns varied across the two age groups of callers reached statistical significance (Table 2). Young adult callers were more likely to report non-family relationship problems (*χ*^2^ = 300.62, *p* < 0.001), while older callers were more likely to have concerns about family relationship problems (*χ*^2^ = 105.66, *p* < 0.001), financial problems (*χ*^2^ = 76.91, *p* < 0.001) and other negative events (*χ*^2^ = 61.16, *p* < 0.001).

As for the younger callers, there was no significant difference in the hopefulness, the severity of depressive symptoms, and psychological distress (*p* > 0.05); while older callers had higher level of hopefulness (*Z* =  − 2.74, *p* = 0.006) and lower level of psychological distress (*Z* =  − 2.02, *p* < 0.043) and depression (*Z* =  − 3.95, *p* < 0.001). The prevalence of SI in 2019 is higher than that in 2020 no matter among the younger (*Z* = 7.14, *p* = 0.008) or the older callers (*Z* = 35.73, *p* < 0.001). Callers were inclined to report depression problems during the pandemic for both the younger (*Z* = 9.07, *p* = 0.003) and older callers (*Z* = 25.54, *p* < 0.001) (Table 3). Callers were less likely to report financial problems during the pandemic for both the younger (*Z* = 7.46, *p* = 0.006) and older callers (*Z* = 11.61, *p* = 0.001).

**Table 3:** Comparison of mental health status and concerns of hotline callers before and during the pandemic between different age groups.

**Variables**	Younger (*n* = 7883)			Older (*n* = 2607)		
	Before pandemic (*n* = 3,756)	During pandemic (*n* = 4,127)		Before pandemic (*n* = 1,184)	During pandemic (*n* = 1,423)	
	**Median (IQR)**	**Median (IQR)**	**Z**	**Median (IQR)**	**Median** **(IQR)**	**Z**
Hopefulness	26 (2,50)	30 (5,50)	−1.91	35 (0,55)	40 (10,60)	−2.74^**^
Psychological distress	80 (70,95)	80 (70,95)	−0.92	85 (70,99)	80 (70,95)	−2.02^*^
Depression severity	68 (54,81)	68 (53,80)	−1.76	69 (52,82)	65 (46,80)	−3.95^***^
	**n (%)**	**n (%)**	*χ* ^**2**^	**n (%)**	**n (%)**	*χ* ^**2**^
Presence of suicidal ideation	2282 (80.4)	2534 (77.6)	7.14^**^	627 (73.2)	631 (60.2)	35.73^***^
	**n (%)**	**n (%)**	*χ* ^**2**^	**n (%)**	**n (%)**	*χ* ^**2**^
Family relationship problems	639 (17.0)	886 (21.5)	25.02^***^	353 (29.8)	401 (28.2)	0.84
Non-family relationship problems	1302 (34.7)	1344 (32.6)	3.88^*^	193 (16.3)	218 (15.3)	0.47
Financial problems	321 (8.5)	285 (6.9)	7.46^**^	188 (15.9)	161 (11.3)	11.61^**^
Work problems	260 (6.9)	270 (6.5)	0.45	84 (7.1)	97 (6.8)	0.08
Depression	823 (21.9)	1023 (24.8)	9.07^**^	229 (19.3)	396 (27.8)	25.54^***^
Other negative events	222 (5.9)	192 (4.7)	6.26^*^	123 (10.4)	126 (8.9)	1.76
Psychiatric problems	622 (16.6)	656 (15.9)	0.64	211 (17.8)	250 (17.6)	0.03

**Notes.**

* *p* < 0.05.

** *p* < 0.01.

*** *p* < 0.001.

Each variable contains missing values, so the sum of the callers of each variable is less than the total number of callers.

Results of multivariable logistic regression analysis revealed that callers during the pandemic were less likely to report SI (OR = 0.69, 95% CI [0.61–0.77]). Callers who were married (OR = 0.78, 95% CI [0.67–0.90]) and had higher education level (OR = 0.79, 95% CI [0.73–0.85]) were less likely to report SI. Callers who were married (OR = 1.21, 95% CI [1.07–1.36]), had higher education level (OR = 1.14, 95% CI [1.08–1.20]) and during the pandemic (OR = 1.13, 95% CI [1.03–1.24]) were inclined to report higher level of hopefulness. Callers who were male (OR = 0.84, 95% CI [0.77–0.91]), had higher education level (OR = 0.92, 95% CI [0.87–0.98]) and during the pandemic (OR = 0.89, 95% CI [0.81–0.98]) were not inclined to report severe psychological distress. While callers who were married were more inclined to report severe psychological distress (OR = 1.23, 95% CI [1.09–1.29]). Callers during the pandemic (OR = 0.81, 95% CI [0.74–0.89]) and had higher education level (OR = 0.91, 95% CI [0.86–0.96]) were less likely to report higher level of depression (Table 4).

**Table 4:** Multivariable logistic regression on the mental health of hotline callers.

	**Having suicidal ideation**		**High hopefulness**		**Severe Psychological distress**		**High depressive score**	
	OR	95% CI	OR	95% CI	OR	95% CI	OR	95% CI
Male								
(ref: female)	0.91	0.82-1.01	1.00	0.92-1.09	0.84^***^	0.77-0.91	1.03	0.95-1.12
Marital status								
(ref: unmarried)	0.78^**^	0.67–0.90	1.21^**^	1.07–1.36	1.23^**^	1.09–1.39	0.97	0.86–1.09
Education years	0.79^***^	0.73–0.85	1.14^***^	1.08–1.20	0.92^**^	0.87–0.98	0.91^**^	0.86–0.96
Pandemic (2020)								
(ref: Pre-pandemic)	0.69^***^	0.61–0.77	1.13^**^	1.03–1.24	0.89^*^	0.81–0.98	0.81^***^	0.74–0.89
Age groups								
(ref: ≥30 years old)	
<30 years old	0.85	0.57–1.26	1.02	0.75–1.39	0.97	0.70–1.35	0.74	0.54–1.01
Age* year interaction								
<30 years old during pandemic	1.52^***^	1.21–1.92	0.89	0.74–1.08	1.07	0.88–1.30	1.15	0.95–1.39

**Notes.**

* *p* < 0.05.

** *p* < 0.01.

*** *p* < 0.001

**Figure 2:**
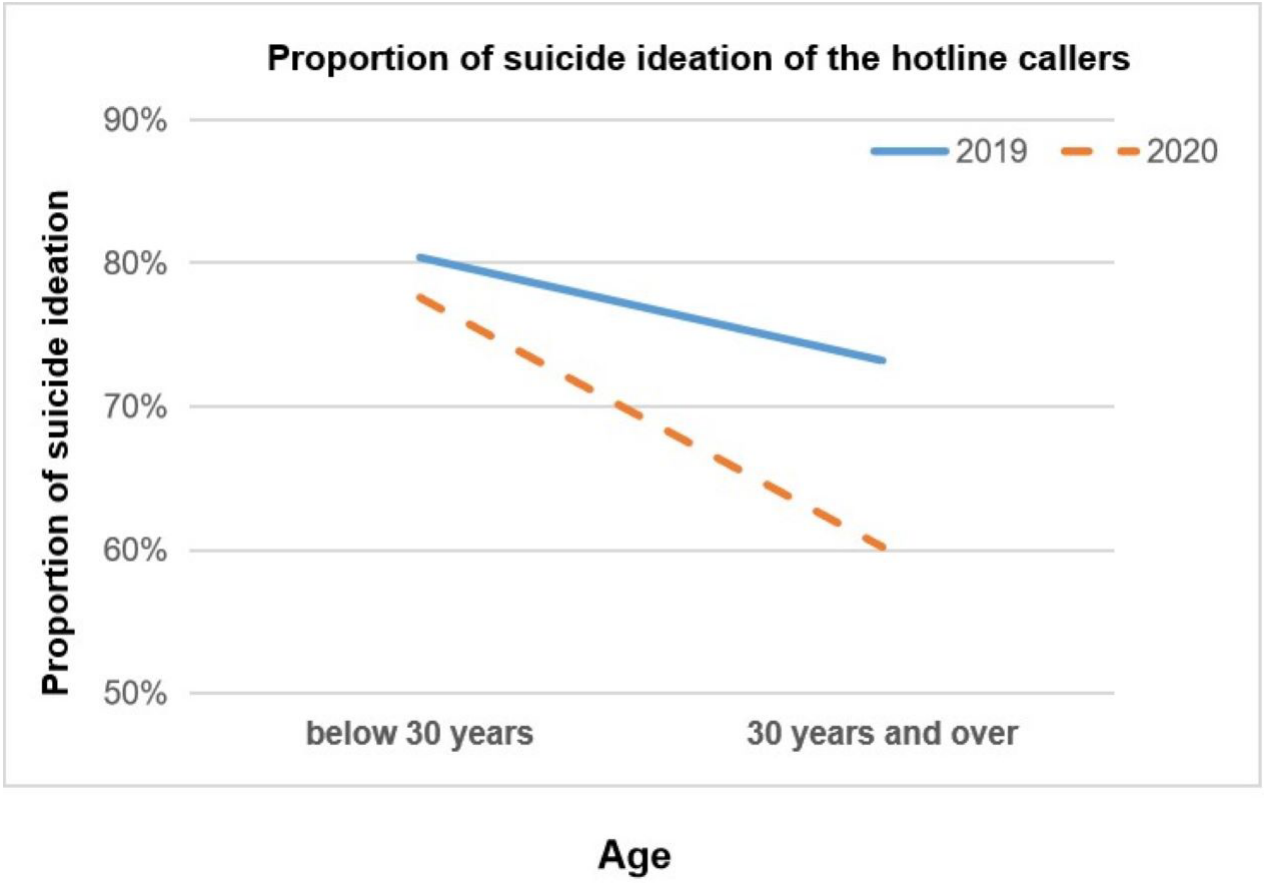
Proportion of suicide ideation of the hotline callers. Comparisons of mental health status of hotline callers during pandemic and before pandemic, moderated by age groups.

The interaction terms of age-group by pandemic period (before and during pandemic) were statistically significant for SI (OR=1.52, 95% CI [1.21–1.92]) (Table 4). It indicated that the change in suicidal ideation is significantly different across age groups. As shown in the Fig. 2, among callers aged 30 or above, the proportion of SI decreased in 2020, compared with same period in 2019, however, the proportion of SI almost unchanged in 2019 and 2020 among the young callers under 30 years old.

## Discussion

Hotlines have emerged as one of the most popular—and safest from a viral transmission standpoint—ways to deliver psychological services during the pandemic (Feinstein et al., 2020; Liu et al., 2020a; Liu et al., 2020b; Wang, Wei & Zhou, 2020). Our study offers an examination of the mental health of callers before and during the early stage of COVID-19 pandemic. Results showed that the overall prevalence of SI among callers was lower during the early stage of pandemic (2020) than before (2019), which was consistent with previous studies on crisis helplines during the COVID-19 pandemic (Brülhart et al., 2021; Zalsman et al., 2021; Batchelor et al., 2021). Meanwhile, different age groups of callers showed different trends in the SI during the pandemic, which suggested that the pandemic seldom caused impact on younger callers, while older adult callers reported less SI during the pandemic, comparing with that in pre-pandemic.

Although there were significant concerns about suicide during the pandemic due to more exposures to suicide risk factors such as social isolation, financial stress, and limited access to treatment (Moutier, 2021; Reger, Stanley & Joiner, 2020; Gunnell et al., 2020), data from 21 countries and regions showed that numbers of suicide in high- and upper-middle-income regions remained stable and even declined in the early month of the pandemic (Pirkis et al., 2021; Hawton et al., 2021). Our study adds to this growing body of research, suggesting that the prevalence of SI among hotline callers during the early stage of pandemic was lower than that in 2019. Hopefulness and depression are important factors related to suicide (Davidson et al., 2009; Bell et al., 2015; Walker, McGee & Druss, 2015). Higher level of hopefulness and lower level of depression of callers during the pandemic, compared with pre-pandemic in our study may contribute to lower prevalence of SI among callers. Though lockdown and financial stress were regarded as suicide risk factors during the pandemic (Moutier, 2021; Reger, Stanley & Joiner, 2020; Gunnell et al., 2020), our study found that callers reported fewer financial problems during the pandemic, besides, lockdown may only occur in specific residential districts which affected by infected cases, which may seldom affect the SI of callers in our study during the early stage of the pandemic.

Relatively better mental health status among older adult callers during the early stage of the pandemic could be attributed to various factors. First, higher level of hopefulness is helpful for improving coping strategies which may in turn decrease the suicidality (Snyder et al., 1996). In the present study, older adult callers reported higher hopefulness during the pandemic than the pre-pandemic, which may be a protective factor for SI and depression. Second, economic recession and unemployment appear to be specific pandemic-related suicide risk factors (Kawohl & Nordt, 2020). However, in our study, older adult callers were less likely to be involved in financial difficulties during the pandemic, which may have decreased their risk Third, older adults may be well-equipped to seek alternative modes of contact, and maintain relationships through the internet and telephone (Chen & Schulz, 2016), which may be useful for dealing with the concerns that social distancing and other lockdown measures may result in greater loneliness and social isolation and worsened mental health among older adults (Goethals et al., 2020; Wu, 2020). Fourth, better emotional regulation, more adaptive coping styles, and experience to deal with stressors were found among older adults (Lee et al., 2019).

Relatively worse mental health status among younger hotline callers during the early stage of the COVID-19 pandemic in our study is consistent with previous studies, in which younger people are at higher risk for mental problems (Liu et al., 2020a; Liu et al., 2020b; Huang & Zhao, 2020; Shi et al., 2020), even suicide risk (Iob, Steptoe & Fancourt, 2020; Czeisler et al., 2020). Worse mental health problems among younger adults may be attributable to the fact that young adults have more exposure to social media (Gao et al., 2020; Zhao & Zhou, 2020; The Lancet, 2019), more psychological pressure from the unemployment financial concerns during the pandemic (Liu et al., 2020a; Liu et al., 2020b; Jia et al., 2020; Gratz et al., 2020). Our study found that younger callers reported more family relationship, and depression problems during the pandemic, suggesting younger callers may be obsessed with economic and unemployment difficulties, which may also explain the result.

In our study, we found that callers had more concerns about their depression problems, which maybe due to the barriers to accessing mental health care (Gunnell et al., 2020), suggesting more convenient access to mental health care, such as remote medical service, delivering medicine by the hospital to patients would be needed during the pandemic. we also found demographic factors were associated with mental health of callers. Unmarried, females and lower educational level were identified as correlated factors for poor mental health outcomes, which was consistent with previous researches (Shi et al., 2020; Lee et al., 2015; Gunnell et al., 2020).

There are several limitations in the present study. First, our study sample included hotline callers who actively seeking psychological help, and older adults may be less likely to use hotlines compared with youngers. Special subgroups of older adults, such as those living in the nursing homes, were not included in our study. Therefore, caution should be used in generalizing our findings to all older adults in China. Second, the year 2020 and 2019 was used as an indicator of exposure to pandemic-related stress or not; however, we did not ask about specific pandemic-related stressors, such as whether callers were exposed to the virus or knew someone affected by the virus. Third, our data relied on callers’ self-report. Future studies may use comprehensive, gold-standard standardized assessments to determine the presence and severity of psychiatric conditions. Fourth, the severity of COVID-19 infection differed regionally, *e.g.*, in Wuhan and other regions in China. However, we could not identify the location of callers via hotline, which limits our ability to investigate this important issue in this study.

## Conclusions

Though our findings implied that during the early stage of the COVID-19 pandemic, callers did not appear more severe mental health problems compared with callers before the pandemic. However, we need to remain vigilant to respond to the long-term mental health effect of the pandemic, as the pandemic is ongoing. The unmarried, females and those with a lower educational level were identified as correlated factors for poor mental health outcomes during the pandemic. Younger callers were more vulnerable to mental health problems compared with the older age groups. Thus, some age-specific mental health prevention should be provided through the hotline during public health emergencies in the future.

## Supplemental Information

10.7717/peerj.13419/supp-1 Supplemental Information 1 sample

10.7717/peerj.13419/supp-2 Supplemental Information 2 code

## Additional Information and Declarations

### Data Availability

The following information was supplied regarding data availability: The raw measurements are available in the Supplementary File.

## References

[ref-1] Altman DG, Bland JM (2003). Interaction revisited: the difference between two estimates. BMJ.

[ref-2] Ammerman BA, Burke TA, Jacobucci R, McClure K (2021). Preliminary investigation of the association between COVID-19 and suicidal thoughts and behaviors in the U.S. Journal of Psychiatric Research.

[ref-3] Batchelor S, Stoyanov S, Pirkis J, Kolves K (2021). Use of kids helpline by children and young people in Australia during the COVID-19 pandemic. Journal of Adolescent Health.

[ref-4] Bell S, Russ TC, Kivimaki M, Stamatakis E, Batty GD (2015). Dose-response association between psychological distress and risk of completed suicide in the general population. JAMA Psychiatry.

[ref-5] Brooks SK, Webster RK, Smith LE, Woodland L, Wessely S, Greenberg N, Rubin GJ (2020). The psychological impact of quarantine and how to reduce it: rapid review of the evidence. The Lancet.

[ref-6] Brülhart M, Klotzbücher V, Lalive R, Reich SK (2021). Mental health concerns during the COVID-19 pandemic as revealed by helpline calls. Nature.

[ref-7] Bryan CJ, Bryan AO, Baker JC (2020). Associations among state-level physical distancing measures and suicidal thoughts and behaviors among U.S. adults during the early COVID-19 pandemic. Suicide and Life-Threatening Behavior.

[ref-8] Caballero-Dominguez CC, Jimenez-Villamizar MP, Campo-Arias A (2020). Suicide risk during the lockdown due to coronavirus disease (COVID-19) in Colombia. Death Studies.

[ref-9] Chan SM, Chiu FK, Lam CW, Leung PY, Conwell Y (2006). Elderly suicide and the 2003 SARS epidemic in Hong Kong. International Journal of Geriatric Psychiatry.

[ref-10] Chen YR, Schulz PJ (2016). The effect of information communication technology interventions on reducing social isolation in the elderly: a systematic review. Journal of Medical Internet Research.

[ref-11] Cheung YT, Chau PH, Yip PS (2008). A revisit on older adults suicides and Severe Acute Respiratory Syndrome (SARS) epidemic in Hong Kong. International Journal of Geriatric Psychiatry.

[ref-12] Cooke JE, Eirich R, Racine N, Madigan S (2020). Prevalence of posttraumatic and general psychological stress during COVID-19: a rapid review and meta-analysis. Psychiatry Research.

[ref-13] Czeisler ME, Lane RI, Petrosky E, Wiley JF, Christensen A, Njai R, Weaver MD, Robbins R, Facer-Childs ER, Barger LK, Czeisler CA, Howard ME, Rajaratnam S (2020). Mental health, substance use, and suicidal ideation during the COVID-19 pandemic—United States, June (2020) 24-30. Morbidity and Mortality Weekly Report.

[ref-14] Davidson CL, Wingate LR, Rasmussen KA, Slish ML (2009). Hope as a predictor of interpersonal suicide risk. Suicide and Life-Threatening Behavior.

[ref-15] Debowska A, Horeczy B, Boduszek D, Dolinski D (2020). A repeated cross-sectional survey assessing university students’ stress, depression, anxiety, and suicidality in the early stages of the COVID-19 pandemic in Poland. Psychological Medicine.

[ref-16] Feinstein RE, Kotara S, Jones B, Shanor D, Nemeroff CB (2020). A health care workers mental health crisis line in the age of COVID-19. Depression and Anxiety.

[ref-17] Gao J, Zheng P, Jia Y, Chen H, Mao Y, Chen S, Wang Y, Fu H, Dai J (2020). Mental health problems and social media exposure during COVID-19 outbreak. PLOS ONE.

[ref-18] Goethals L, Barth N, Guyot J, Hupin D, Celarier T, Bongue B (2020). Impact of home quarantine on physical activity among older adults living at home during the COVID-19 pandemic: qualitative interview study. JMIR Aging.

[ref-19] Gratz KL, Tull MT, Richmond JR, Edmonds KA, Scamaldo KM, Rose JP (2020). Thwarted belongingness and perceived burdensomeness explain the associations of COVID-19 social and economic consequences to suicide risk. Suicide and Life-Threatening Behavior.

[ref-20] Gunnell D, Appleby L, Arensman E, Hawton K, John A, Kapur N, Khan M, O’Connor RC, Pirkis J (2020). Suicide risk and prevention during the COVID-19 pandemic. The Lancet Psychiatry.

[ref-21] Hawton K, Casey D, Bale E, Brand F, Ness J, Waters K, Kelly S, Geulayov G (2021). Self-harm during the early period of the COVID-19 pandemic in England: comparative trend analysis of hospital presentations. The Journal of Affective Disorders.

[ref-22] Huang Y, Zhao N (2020). Generalized anxiety disorder, depressive symptoms and sleep quality during COVID-19 outbreak in China: a web-based cross-sectional survey. Psychiatry Research.

[ref-23] Iob E, Steptoe A, Fancourt D (2020). Abuse, self-harm and suicidal ideation in the UK during the COVID-19 pandemic. British Journal of Psychiatry.

[ref-24] Jia R, Ayling K, Chalder T, Massey A, Broadbent E, Coupland C, Vedhara K (2020). Mental health in the UK during the COVID-19 pandemic: cross-sectional analyses from a community cohort study. BMJ Open.

[ref-25] Kawohl W, Nordt C (2020). COVID-19, unemployment, and suicide. The Lancet Psychiatry.

[ref-26] Lee EE, Depp C, Palmer BW, Glorioso D, Daly R, Liu J, Tu XM, Kim HC, Tarr P, Yamada Y, Jeste DV (2019). High prevalence and adverse health effects of loneliness in community-dwelling adults across the lifespan: role of wisdom as a protective factor. International Psychogeriatryics.

[ref-27] Lee S, Leung CM, Kwok KP, Lam NK (2015). A community-based study of the relationship between somatic and psychological distress in Hong Kong. Transcultural Psychiatry.

[ref-28] Li XY, Phillips MR, Zhang YP, Wang ZQ (2007). Development and validity of a diagnostic screening instrument for depression. Chinese Journal of Nervous and Mental Diseases.

[ref-29] Liu X, Luo WT, Li Y, Li CN, Hong ZS, Chen HL, Xiao F, Xia JY (2020a). Psychological status and behavior changes of the public during the COVID-19 epidemic in China. Infectious Diseases of Poverty.

[ref-30] Liu S, Yang L, Zhang C, Xiang YT, Liu Z, Hu S, Zhang B (2020b). Online mental health services in China during the COVID-19 outbreak. The Lancet Psychiatry.

[ref-31] Luo M, Guo L, Yu M, Jiang W, Wang H (2020). The psychological and mental impact of coronavirus disease 2019 (COVID-19) on medical staff and general public—a systematic review and meta-analysis. Psychiatry Research.

[ref-32] Moutier C (2021). Suicide prevention in the COVID-19 era: transforming threat into opportunity. JAMA Psychiatry.

[ref-33] O’Connor RC, Wetherall K, Cleare S, McClelland H, Melson AJ, Niedzwiedz CL, O’Carroll RE, O’Connor DB, Platt S, Scowcroft E, Watson B, Zortea T, Ferguson E, Robb KA (2020). Mental health and well-being during the COVID-19 pandemic: longitudinal analyses of adults in the UK COVID-19 Mental Health & Wellbeing study. British Journal of Psychiatry.

[ref-34] Phillips MR, Shen Q, Liu X, Pritzker S, Streiner D, Conner K, Yang G (2007). Assessing depressive symptoms in persons who die of suicide in mainland China. Journal of Affective Disorders.

[ref-35] Pirkis J, John A, Shin S, DelPozo-Banos M, Arya V, Analuisa-Aguilar P, Appleby L, Arensman E, Bantjes J, Baran A, Bertolote JM, Borges G, Brecic P, Caine E, Castelpietra G, Chang SS, Colchester D, Crompton D, Curkovic M, Deisenhammer EA, Du C, Dwyer J, Erlangsen A, Faust JS, Fortune S, Garrett A, George D, Gerstner R, Gilissen R, Gould M, Hawton K, Kanter J, Kapur N, Khan M, Kirtley OJ, Knipe D, Kolves K, Leske S, Marahatta K, Mittendorfer-Rutz E, Neznanov N, Niederkrotenthaler T, Nielsen E, Nordentoft M, Oberlerchner H, O’Connor RC, Pearson M, Phillips MR, Platt S, Plener PL, Psota G, Qin P, Radeloff D, Rados C, Reif A, Reif-Leonhard C, Rozanov V, Schlang C, Schneider B, Semenova N, Sinyor M, Townsend E, Ueda M, Vijayakumar L, Webb RT, Weerasinghe M, Zalsman G, Gunnell D, Spittal MJ (2021). Suicide trends in the early months of the COVID-19 pandemic: an interrupted time-series analysis of preliminary data from 21 countries. The Lancet Psychiatry.

[ref-36] Reger MA, Stanley IH, Joiner TE (2020). Suicide mortality and coronavirus disease 2019—a perfect storm?. JAMA Psychiatry.

[ref-37] Shi L, Lu ZA, Que JY, Huang XL, Liu L, Ran MS, Gong YM, Yuan K, Yan W, Sun YK, Shi J, Bao YP, Lu L (2020). Prevalence of and risk factors associated with mental health symptoms among the general population in China during the coronavirus disease 2019 pandemic. JAMA Network Open.

[ref-38] Snyder CR, Sympson SC, Ybasco FC, Borders TF, Babyak MA, Higgins RL (1996). Development and validation of the State Hope Scale. Journal of Personality and social psychology.

[ref-39] The Lancet (2019). Social media, screen time, and young people’s mental health. The Lancet.

[ref-40] Tong Y, Conner KR, Wang C, Yin Y, Zhao L, Wang Y, Liu Y (2020). Prospective study of association of characteristics of hotline psychological intervention in 778 high-risk callers with subsequent suicidal act. Australian and New Zealand Journal of Psychiatry.

[ref-41] Walker ER, McGee RE, Druss BG (2015). Mortality in mental disorders and global disease burden implications: a systematic review and meta-analysis. JAMA Psychiatry.

[ref-42] Wang J, Wei H, Zhou L (2020). Hotline services in China during COVID-19 pandemic. Journal of Affective Disorders.

[ref-43] Wu B (2020). Social isolation and loneliness among older adults in the context of COVID-19: a global challenge. Global Health Research and Policy.

[ref-44] Zalsman G, Levy Y, Sommerfeld E, Segal A, Assa D, Ben-dayan L, Valevski A, Mann JJ (2021). Suicide-related calls to a national crisis chat hotline service during the COVID-19 pandemic and lockdown. Journal of Psychiatric Research.

[ref-45] Zhao L, Li Z, Tong Y, Wu M, Wang C, Wang Y, Liu NH (2021). Comparisons of characteristics between psychological support hotline callers with and without COVID-19 related psychological problems in China. Frontiers in Psychiatry.

[ref-46] Zhao N, Zhou G (2020). Social media use and mental health during the COVID-19 Pandemic: moderator role of disaster stressor and mediator role of negative affect. Applied Psychology: Health and Well-Being.

[ref-47] Zortea TC, Brenna C, Joyce M, McClelland H, Tippett M, Tran MM, Arensman E, Corcoran P, Hatcher S, Heisel MJ, Links P, O’Connor RC, Edgar NE, Cha Y, Guaiana G, Williamson E, Sinyor M, Platt S (2020). The impact of infectious disease-related public health emergencies on suicide, suicidal behavior, and suicidal thoughts. Crisis.

